# Cats and *Toxoplasma gondii*: A systematic review and meta-analysis in Iran

**DOI:** 10.4102/ojvr.v82i1.823

**Published:** 2015-04-30

**Authors:** Mohammad T. Rahimi, Ahmad Daryani, Shahabeddin Sarvi, Azar Shokri, Ehsan Ahmadpour, Saeed H. Teshnizi, Azade Mizani, Mahdi Sharif

**Affiliations:** 1 Toxoplasmosis Research Centre, Mazandaran University of Medical Sciences, Sari, Iran; 2 Department of Parasitology and Mycology, Sari Medical School, Mazandaran University of Medical Sciences, Sari, Iran; 3 Infectious and Tropical Diseases Research Centre, Tabriz University of Medical Sciences, Tabriz, Iran; 4 Paramedical School, Hormozgan University of Medical Science, Bandar Abbas, Iran

## Abstract

*Toxoplasma gondii* is a cosmopolitan zoonotic intracellular coccidian of the phylum Apicomplexa infecting warm-blooded animals and human beings. This protozoan causes a significant public health problem in humans and imposes considerable economic losses and damages to husbandry industries. The final host, cats, accounts for all of these significant burdens. Hence the present study was designed to analyse and review the overall prevalence rate of *T. gondii* infection in cats in Iran for the first time. In the present study data collection (published and unpublished papers, abstracts of proceedings of national parasitology congresses and dissertations) was systematically undertaken on electronic databases including PubMed, Google Scholar, Ebsco, Science Direct, Scopus, Magiran, Irandoc, IranMedex and Scientific Information Database. A total of 21 studies from 1975 to 2013 reporting prevalence of Toxoplasma infection in cats from different areas in Iran met the eligibility criteria. The pooled proportion of toxoplasmosis using the random-effect model amongst cats was estimated at 33.6% (95% confidence interval [CI] 22.05–46.41). The prevalence rate of cat toxoplasmosis in various regions of Iran ranged from 1.2% to 89.2%. Firstly, this study establishes a crude prevalence rate of *T. gondii* infection in cats. Secondly, it discusses the role of significant risk factors including sex, age and being either household or stray cats, in the epidemiology of the disease. Furthermore, the current study determines gaps and drawbacks in the prior studies that are useful to keep in mind to assist in designing more accurate investigations in future.

## Introduction

*Toxoplasma gondii*, the causative agent of toxoplasmosis, is an obligate intracellular parasite which belongs to the phylum Apicomplexa that infects all species of warm-blooded animals (Flegr *et al.* 2003). In humans it is considered to be one of the most common parasites, based on serological investigations that estimate that up to a third of the world's population has been exposed to this widespread zoonotic agent. The overall seroprevalence rate of toxoplasmosis amongst the general population in Iran is 39.3% (95% confidence interval [CI] 33.0% – 45.7%) (Bahrami *et al.* 2011; Daryani *et al.* 2014; Dubey & Jones 2008; Sharif *et al.* 2007).

Even though the majority of toxoplasmosis cases in immune-competent individuals are either asymptomatic or mild, first exposure to *T. gondii* during pregnancy can lead to transplacental transmission to the embryo, with serious pathological signs including hydrocephalus, microcephaly, blindness, abortion and death of the foetus (Dunn *et al.* 1999; Havelaar, Kemmeren & Kortbeek 2007). In addition, *T. gondii* is considered to be an opportunistic and life-threatening parasite in immune-compromised groups, encompassing those with HIV and AIDS, cancer and organ transplant recipients who receive immunosuppressive drugs (Tenter, Heckeroth & Weiss 2000).

Felids play a pivotal role for *T. gondii* as definitive hosts, and interestingly are known as the only final hosts that produce oocysts in their faeces, contaminating soil, food and water (Cenci-Goga *et al.* 2011; Dubey 2004). Even though the final host excretes oocysts for a short period of time (only 1–2 weeks), millions of oocysts may be excreted. Oocysts can survive in the environment for several months and are noticeably resistant to freezing, drying and disinfectants, whereas they are not heat-resistant and are destroyed at 70 °C for 10 min (Dubey & Beattie 1988; Dubey & Jones 2008).

Infection both in the definitive and the intermediate host usually occurs either through ingestion of infected tissue cysts or oocysts. Felids are more likely to shed oocysts followed by ingestion of tissue cysts rather than oocysts. Surprisingly, a cat must ingest at least 1000 oocysts in order to develop an infection, although ingestion of just one bradyzoite is enough for a cat to acquire *T. gondii* infection (Dubey 2008).

Clinical manifestations of cats infected with *T. gondii* include depression, anorexia and fever, followed by peritoneal effusion, hypothermia, icterus and dyspnoea. Moreover, some other symptoms of toxoplasmosis are diarrhoea, weight loss, muscle hyperaesthesia, fever, anorexia, seizures, ataxia, pancreatitis and anterior or posterior uveitis (Dubey & Lappin 2006). Furthermore, the coccidian phase of the entero-epithelial cycle is seen solely in the definitive feline host. The extra-intestinal development of *T. gondii* is the same for all hosts, including all warm-blooded vertebrates (Dubey 2005). The disease is important both in the medical and veterinary fields. Toxoplasmosis causes significant economic losses and damages to animal husbandry due to stillbirths and neonatal mortality in sheep and goats (Dubey & Beattie 1988; Hartley & Marshall 1957).

In spite of the need, currently there is no effective vaccine, even though many efforts have been conducted to develop a vaccine and are ongoing. Furthermore, no approved treatment exists for clinical toxoplasmosis in cats. Drugs including pyrimethamine, sulphonamides, trimethoprim and clindamycin, either alone or in combination, that have been prescribed to treat cats with clinical toxoplasmosis have shown varied results (Dabritz *et al.* 2007).

Cats have a key and crucial role in the epidemiology of toxoplasmosis, so expanding the basic knowledge about *T. gondii* infection in cats is a matter of importance. It is worth mentioning that epidemiological investigations are still the most useful method for evaluating the status of *T. gondii* infection. Despite the multitude of publications on toxoplasmosis in cats from Iran, there is no systematic review and meta-analysis that can describe the status of toxoplasmosis in the final host in this country. Therefore the objective of the current systematic review and meta-analysis was to determine the weighted prevalence of *T. gondii* infection and describe the epidemiological features of infection in cats in Iran.

## Research method and design

### Database search

**FIGURE 1:**
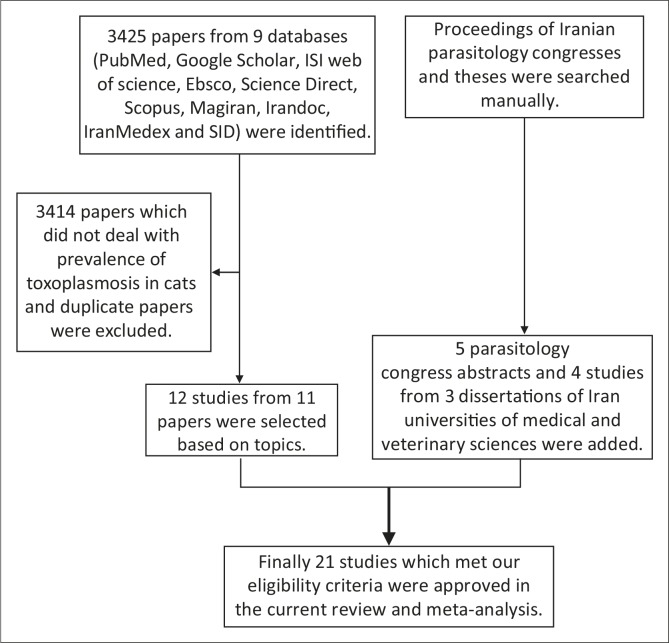
Flowchart describing the study design process.

To gather information a precise and comprehensive search was performed on all scientific publications (full texts and abstracts) from February to April in 2013 (Figure 1). The following nine databases were included: five English databases (PubMed, Google Scholar, Ebsco, Science Direct and Scopus) and four Persian databases (Magiran, Irandoc, IranMedex and the Scientific Information Database [SID]). In addition to published articles, dissertations and all proceedings of national parasitology congresses held in Iran from 1975 to 2013 were carefully evaluated. In order to avoid missing any articles, whole references of papers were also meticulously checked.

The search terms used alone or in combination were ‘*Toxoplasma gondii*’, ‘toxoplasmosis’, ‘*Toxoplasma* infection’, ‘animal toxoplasmosis’, ‘cat’, ‘feline’, ‘epidemiology’, ‘prevalence’, ‘Iran’ and ‘anti-*Toxoplasma* antibodies’. Data collection was limited to items in English and Persian.

### Data collection

All cross-sectional studies carried out to estimate the prevalence of toxoplasmosis, diagnosed by different methods using serological, molecular and parasitological tests on cats, were included. Repetitive papers were excluded. The data that were collected for the current study were as follows: year of publication, first author, study areas, sample size, number of males and females, prevalence rate, age of samples, diagnostic tests, time of the study, and involving either domestic or stray cats. For this purpose a data extraction form was used.

The quality of the meta-analysis was evaluated using the Strengthening the Reporting of Observational Studies in Epidemiology (STROBE) checklist, which included 22 items that we considered essential for good reporting of observational studies. These items were related to the article's title, abstract, introduction, methods, results and discussion sections. Scores under 7.75 were considered to indicate bad quality, of 7.76–15.5 low quality, 15.6–23.5 moderate and more than 23.6 high quality (Von Elm *et al.* 2007).

### Statistical methods

The crude and the weighted prevalence estimates as well as the 95% CI for each study that was included were calculated. A forest plot was used to visualise the heterogeneity amongst studies. The heterogeneity was expected in advance, and statistical methods such as I^2^ and Cochrane's Q test (with a significance level of *p* < 0.1) were used to quantify variations. For the purpose of meta-analysis we assumed that the included studies were random samples from the populations under study, and a random-effect model was employed. Meta-regression and subgroup analyses were employed to assess the cause of heterogeneity amongst the selected studies, and Egger's regression test and funnel plotting were used to evaluate publication bias.

Proportions of individual studies and overall prevalence were presented using forest plots. The meta-analysis was performed using the trial version of StatsDirect statistical software (http://www.statsdirect.com).

## Results

From the nine databases, 21 studies met the eligibility criteria and were included in the current systematic review and meta-analysis. A mean score of 16.8 using the STROBE checklist (Von Elm *et al.* 2007) was obtained for the 21 studies that were analysed.

A flowchart depicts the study design process (Figure 1). A total number of 2145 cats was examined for toxoplasmosis from 1975 to 2013 in different areas of Iran, and 662 cases were diagnosed as positive using different diagnostic methods (Tables 1 and 2).

**TABLE 1:** Publications on cat toxoplasmosis included for meta-analysis.

City	Province	Diagnostic method	Cut-off	Total individuals	Positive individuals	Prevalence (%)	Year	Authors
Different cities in Iran	Different provinces in Iran	LAT	-	111	19	17.1	1983	Ghorbani
Tehran	Tehran	DAT	1:20	102 stray	91	89.2	1992	Sayyed Tabaei
Ahvaz	Khozestan	DAT	1:20	101 stray	60	59.4	1993	Hoghooghi-Rad and Afraa
Tabriz	East Azerbaijan	SFT	-	100 stray	36	36	1996	Jamali
Tehran	Tehran	IFA	1:32	50 domestic, 50 stray	63	63	2006	Haddadzadeh *et al*.
Kashan	Isfahan	IFA	1:20	50 stray	43	86	2007	Hooshyar *et al*.
Shiraz	Fars	IFA	-	100 stray	11	11	2008	Tahmtan
Sari	Mazandaran	LAT	≥ 1:1	100 stray	40	40	2009	Sharif **et al.**
Isfahan	Isfahan	IFA	1:80	100 stray	32	32	2010	Saljoghiyan
Tehran	Tehran	ICT	-	65 domestic	2	3	2010	Skoeizade
Kerman	Kerman	MAT	≥ 1:20	70 domestic, 70 stray	32	22.8	2010	Akhtardanesh **et al.**
Shiraz	Fars	MAT	1:20	29 stray	24	82.2	2011	Pirzad
Shiraz	Fars	PCR	-	29 stray	20	69	2011	Pirzad
Urmia	West Azerbaijan	MAT	1:20	100 domestic, 30 stray	27	35.3	2011	Raeghi and Sedeghi
Ahvaz	Khozestan	ICT	-	198 domestic	49	24.7	2011	Mosallanejad **et al.**
Kerman	Kerman	IFA	≥ 1:16	108 stray	3	2.7	2012	Derakhshan and Mousavi

Note: Please see the full reference list of the article, Rahimi, M.T., Daryani, A., Sarvi, S., Shokri, A., Ahmadpour, E., Mizani, A. *et al*., 2015, ‘Cats and *Toxoplasma gondii*: A systematic review and meta-analysis in Iran’, *Onderstepoort Journal of Veterinary Research* 82(1), Art. #823, 10 pages. http://dx.doi.org/10.4102/ojvr.v82i1.823, for more information.

LAT, latex agglutination test; DAT, direct agglutination test; SFT, Sabin and Feldman Test; IFA, indirect immunofluorescent assay; ICT, immunochromatography test; MAT, modified agglutination test; PCR, polymerase chain reaction.

**TABLE 2:** Studies of cat toxoplasmosis included for meta-analysis based on stool examination.

City	Province	Diagnostic method	Total individuals	Positive individuals	Prevalence (%)	Year	Authors
Tehran	Tehran	Wet smear	170 domestic	21	12.3	1975	Saatara
Khorasan	Khorasan	Flotation	82 stray	1	1.2	2000	Razmi
Zanjan	Zanjan	Flotation	100 stray	42	42	2009	Esmaeilzadeh **et al.**
Tabriz	East Azerbaijan	Flotation	100	43	43	2010	Farhang
Urmia	West Azerbaijan	Flotation	100 domestic, 30 stray	3	2.3	2011	Raeghi and Sedeghi

Note: Please see the full reference list of the article, Rahimi, M.T., Daryani, A., Sarvi, S., Shokri, A., Ahmadpour, E., Mizani, A. *et al*., 2015, ‘Cats and *Toxoplasma gondii*: A systematic review and meta-analysis in Iran’, *Onderstepoort Journal of Veterinary Research* 82(1), Art. #823, 10 pages. http://dx.doi.org/10.4102/ojvr.v82i1.823, for more information.

**FIGURE 2:**
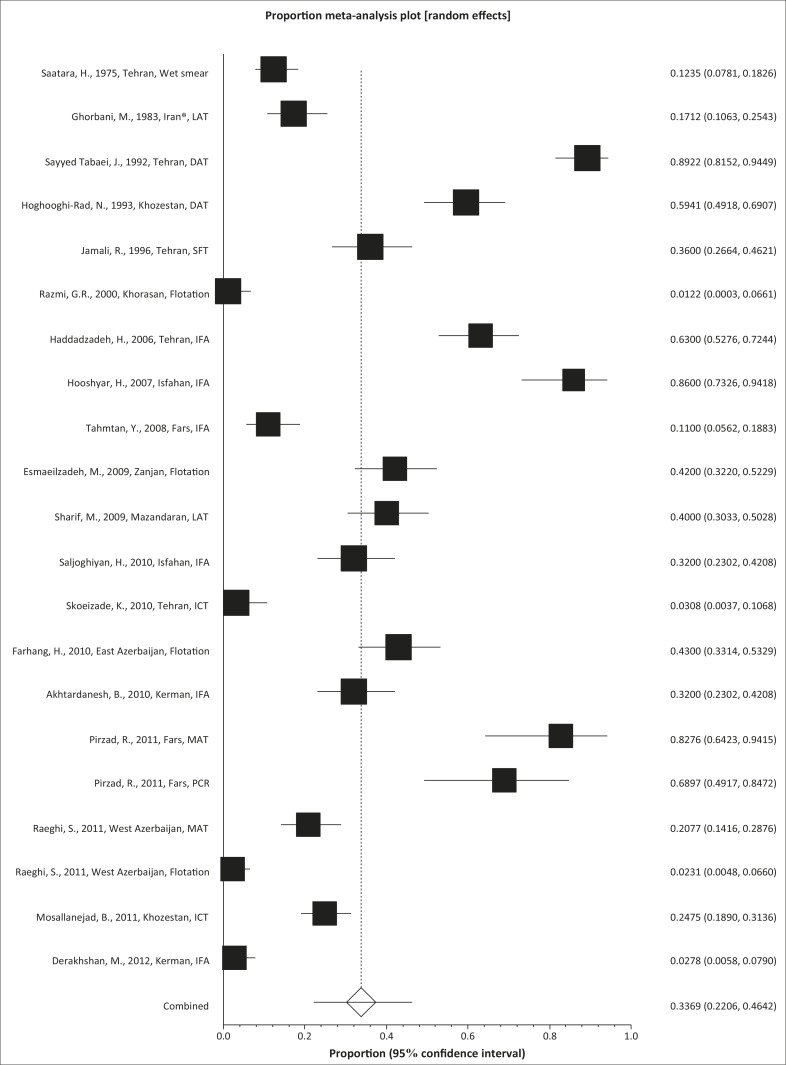
Forest plot diagram of the current systematic review and meta-analysis.

During a period of 39 years, nine different types of diagnostic methods were employed to evaluate *T. gondii* infection in cats, as follows: the modified agglutination test (MAT), direct agglutination test (DAT), indirect immunofluorescent assay (IFA), latex agglutination test (LAT), immunochromatography test (ICT) and Sabin and Feldman Test (SFT), wet smear, flotation and polymerase chain reaction (PCR). The most frequently used diagnostic method for *T. gondii* assessment in cats in Iran was the IFA (5 studies), followed by flotation (4 studies), MAT (3 studies), LAT (2 studies), DAT (2 studies), ICT (2 studies), SFT, PCR and wet smear (1 study for each). The pooled proportion of toxoplasmosis, using the random-effect model, amongst cats in Iran over the 39-year period was estimated at 33.6% (95% CI 22.05–46.41) and a forest plot diagram of the current study was drawn (Figure 2). A wide variation was observed in the prevalence estimates of different studies (Q statistic = 742.3, *df* = 20, *p* < 0.0001 and I² = 99%).

**FIGURE 3:**
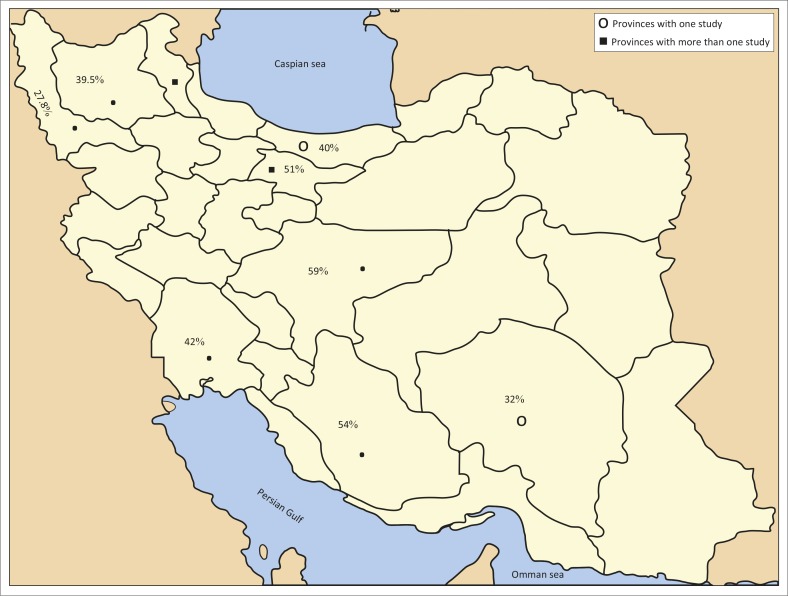
Prevalence of toxoplasmosis in cats in different provinces.

The prevalence rate of cat toxoplasmosis in various regions of Iran was between 1.2% and 89.2% in Khorasan and Tehran respectively. The prevalence rate of toxoplasmosis in cats in different parts of Iran is shown in Figure 3. Amongst the studies included, only three studies compared stray cats with domestic cats for toxoplasmosis (Akhtardanesh *et al.* 2010; Haddadzadeh *et al.* 2006; Raeghi & Sedeghi 2011). They found a statistically significant difference in the number of positive stray cats and domestic cats. The results based on age distribution were mentioned in 6 out of 21 surveys. A significantly higher prevalence rate of *T. gondii* was observed in older animals as compared with younger ones. Regarding sex, just two studies reported a significant difference, with male cats showing a higher prevalence rate of toxoplasmosis than females (Farhang 2010; Raeghi & Sedeghi 2011). The source of faeces in all the studies was the rectum of the examined cats.

**FIGURE 4:**
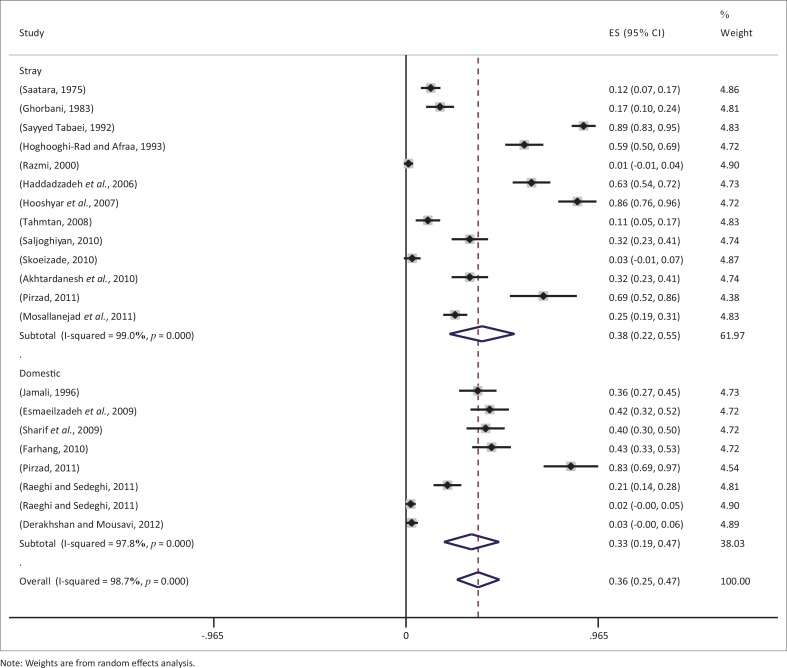
Prevalence of seropositivity in terms of type of cats (stray or domestic).

Note: Weights are from random effects analysis.

Results of heterogeneity of meta-analysis for two groups (stray and domestic cats) showed that they were not homogeneous (*p* < 0.0001). Overall seroprevalence rates for stray and domestic cats were 0.38 (95% CI 0.22–0.55) and 0.33 (95% CI 0.19–0.47) respectively, and the pooled estimate was 0.36 (95% CI 0.25–0.47) (Figure 4).

**FIGURE 5:**
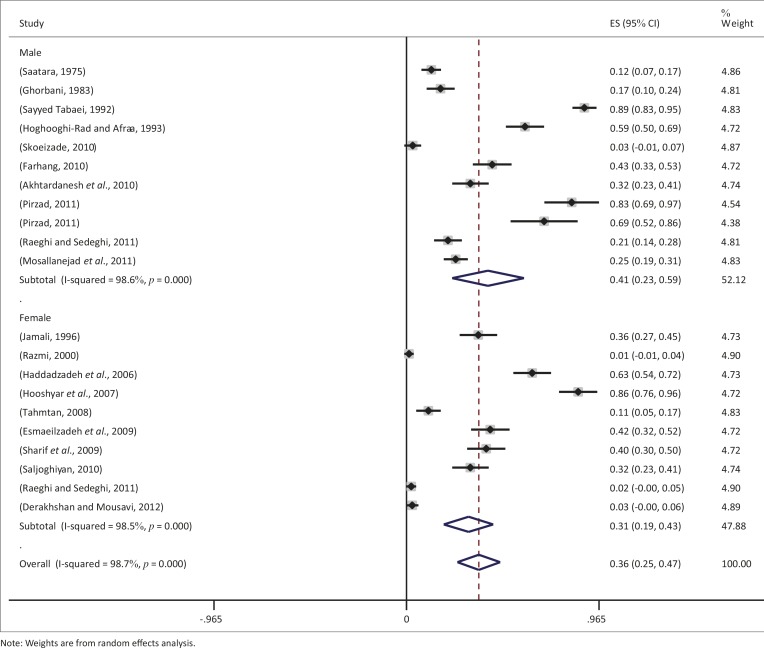
Prevalence of seropositivity by sex of cats.

Note: Weights are from random effects analysis.

There was a significant difference between sub-groups of sexes (*p* < 0.0001), and random meta-analysis showed that seroprevalence rates of toxoplasmosis in males and females were 0.21 (95% CI 0.23–0.59) and 0.31 (95% CI 0.19–0.43), respectively, and the pooled estimate was 0.36 (95% CI 0.25–0.47) (Figure 5).

Differences between sub-groups of ages (juvenile and adult) were significant (*p* < 0.0001) in only two studies, and random meta-analysis showed seroprevalence rates of toxoplasmosis in juveniles and adults to be 0.545 (95% CI 0.540–0.550) and 0.392 (95% CI 0.286–0.562) respectively. The pooled estimate was 0.481 (95% CI 0.386–0.788) (Table 3).

**TABLE 3:** Prevalence of seropositivity by age of cats.

Study	No.	ES*	95% confidence interval	Weight (%)	Authors
Juvenile	100	0.545	0.540, 0.550	50	Sharif *et al.* (2009)
Adult	198	0.392	0.286, 0.562	50	Mosallanejad, *et al*.
Total	298	0.481	0.386, 0.788	100	D+L pooled ES

Heterogeneity χ^2^ = 41571.85 (*df* = 1) *p* = 0.000.

I^2^ (variation in ES attributable to heterogeneity) = 100.0%.

Estimate of between-study variance τ^2^ = 0.1285.

**FIGURE 6:**
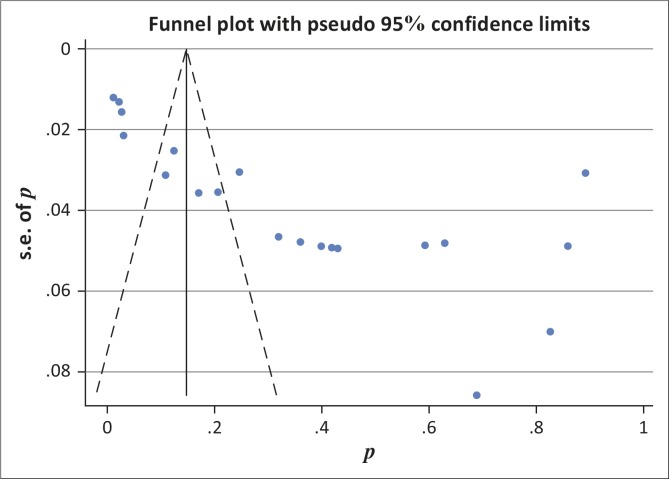
Funnel plots from Egger.

Egger's regression test and a funnel plot were carried out for assessment of publication bias, and results showed that publication bias was significant (Figure 6).

Five studies employed stool examination on 582 cats and 110 cases (18.9%) were reported as positive.

## Discussion

The current study is the first systematic review and meta-analysis of cat toxoplasmosis in Iran. It provides valuable data about the prevalence of toxoplasmosis in cats from 1975 to 2013. The overall prevalence rate of toxoplasmosis amongst cats in Iran was estimated to be 33.6%. The worldwide seroprevalence of toxoplasmosis in domestic cats (*Felis catus*) was estimated to be 30% – 40% (Dubey & Beattie 1988), and our findings roughly correspond to this range. Besides, previous studies have shown that the prevalence rates of *T. gondii* antibodies in cat populations vary greatly, from 0% to 100%, and depend on particular criteria including age, method of survey, number of animals studied and geographical area (Dubey 2005; Dubey & Beattie 1988).

Several surveys have been performed on cat toxoplasmosis in Iran's neighbouring countries. An investigation on anti-*T. gondii* antibodies in 99 cats in Ankara, Turkey, using SFT and IFA showed prevalences of 40.3% and 34.3% respectively (Özkan *et al*. 2008), which is in close alignment with the present study. In another survey in Nigde, Turkey, the prevalence of antibodies to *T. gondii* employing SFT in stray cats was reported to be 76.4% (Karatepe *et al.* 2009). In Saudi Arabia the prevalence rate of *T. gondii* antibodies in cats was determined to be 15.2% using the indirect haemagglutination (IHA) test (Hossain *et al.* 1986).

A study on stray and domestic cats using LAT in Lahore, Pakistan, showed that 56% of cases were seropositive for *T. gondii* (domestic cats 48% and stray cats 64%) (Shahzad *et al.* 2006). In addition, the seroprevalence rate of *T. gondii* infection in cats was reported to be 60% in Faisalabad, Pakistan using LAT (Ahmad *et al.* 2001). The infection rates in cats in Lebanon and Iraq were reported to be 78.1% and 100%, respectively (Deeb, Sufan & Digiacomo 1985; Khairy, Alaa & Ahmad 2010). The findings of the current study are in agreement with those of Dubey (1998), who reported a 30% – 80% seroprevalence rate in cats in the United States of America (USA).

A high prevalence of toxoplasmosis of cats in some areas may be due to the following factors: humid and temperate climate; absence of routine treatment for feline toxoplasmosis; and a considerable abundance of cats. It was confirmed that there is conformity between climate and the prevalence rate of toxoplasmosis, and regional prevalence varied in conformity with different climates. It is usually more prevalent in warm, humid climates and at lower altitudes compared to cold or dry districts. This fact is associated with longer viability of *T. gondii* oocysts in a warm and humid environment (Tutuncu *et al.* 2003). Sporulated oocysts of *T. gondii* can persist in the environment (particularly in moist shaded soil or sand) for several months owing to their resistance to variable environ­mental conditions (Dubey & Beattie 1988; Webster 2001).

In the USA the prevalence rate in the drier southwest, including New Mexico, Utah and Arizona, was lower (16.1%) than in humid climates such as Hawaii (59.2%), in accordance with the abovementioned climate template for *T. gondii* infection (Dubey & Jones 2008). In contrast, in some areas of Iran, such as Mazandaran province, the findings are not in accordance with that climate template.

Iran's climate ranges from arid or semi-arid to subtropical along the Caspian coast and in the northern forests. In the north of the country temperatures seldom decrease below freezing in winter, and the area usually remains humid for the rest of the year. In summer the temperature rarely exceeds 29 °C (84.2 °F) (Modarres & De Paulo Rodrigues Da Silva 2007).

In the past different diagnostic tests were used in *T. gondii* surveys in cats. IFA was the most frequently employed test for the diagnosis of cat toxoplasmosis. This test was introduced in 1992 and considered a more reliable test than other serological diagnostic methods due to its significant sensitivity and specificity. This method is a relatively simple assay for evaluating the infection of animals, and also is particularly useful for screening a large number of specimens, which may explain why most studies used this method (Chejfec 1999; De La Luz Galvan-Ramirez *et al.* 2012; Silva *et al.* 2002).

In this systematic review and meta-analysis sex, age and type of cat (stray or domestic) can be probable causes of homogeneity, as well as publication bias. The role of risk factors including sex, age and being stray or domestic in the prevalence of toxoplasmosis is undeniable. In the current review there was a statistically significant difference between sexes (*p* < 0.05), and a higher infection rate of toxoplasmosis was seen in male cats compared to females. This may be related to lifestyle, as male cats have more of a tendency to wander and thus more access to contaminated sources; spending more time outdoors may increase their exposure to infection.

The age of animals is another major factor in prevalence of toxoplasmosis. Since young animals were less infected than older ones, it is expected that with increasing age exposure to *T. gondii* infection also increases (Miró *et al.* 2004). In Iran a significant relationship was observed between age at sampling and prevalence rate of toxoplasmosis amongst cats. These findings were in accordance with the results of some studies in other countries, which reported an increased prevalence of toxoplasmosis in older compared to younger cats (Frenkel *et al.* 1995; Maruyama *et al.* 2003; Vollaire, Radecki & Lappin 2005). It has been mentioned that newborn kittens are more dangerous than adult cats for transmission of this infectious disease, since they excrete oocysts for 1–2 weeks. On the other hand, kittens infected transplacentally or via milk exhibit more severe symptoms and frequently die due to pulmonary or hepatic disease (Buxton & Rodger 2008).

In the present study, Haddadzadeh *et al.* (2006), Akhtardanesh *et al.* (2010) and Raeghi and Sedeghi (2011) showed that the prevalence of toxoplasmosis in stray cats was noticeably higher than in domestic cats. In general, stray cats are more prone to *T. gondii* infection compared to household cats. The findings confirmed that stray cats have a tendency to have a higher prevalence rate than cats kept indoors. An acceptable justification for this fact might be that stray cats could acquire the infection through catching wild rodents, birds and reptiles, raw food scraps and so on. Cat owners can decrease their cats’ exposure risk of acquiring *T. gondii* infection by keeping their cats indoors and also not feeding them uncooked meat and milk. In addition, the food source of the animal is significant in transmission and also for completion of the lifecycle of the parasite (Dubey *et al.* 2006). Integrated control strategies and measures should be considered to prevent and control toxoplasmosis in both stray and household cats, which will have important implications for the control of toxoplasmosis in humans and other important intermediates such as sheep, goats and cattle.

## Limitations of the study

The collected data are limited just to 10 out of 31 provinces, and there is a paucity of data for cat toxoplasmosis in the majority of provinces of Iran. Although many efforts have been made to determine the prevalence of toxoplasmosis in cats in different parts of Iran, some gaps in prior studies were evident.

### Recommendations

Not enough attention was paid during sampling to the role of major factors including sex, age, and being stray or companion cats, despite their key role in the epidemiology of the disease. Therefore, considering all of the abovementioned parameters is necessary in order to overcome these shortcomings in future. The present study will form the basis of further studies that will enable us to deepen our knowledge of the epidemiology of *T. gondii*.

## Conclusion

Based on the current results, stray cats are probably the major source of *T. gondii* infection in Iran. This study accentuates some valuable and interesting points: the prevalence rate of toxoplasmosis in cats in Iran is high (33.6%), and this considerable infection rate of final hosts can be considered a potential danger to public health and animals due to high contamination of the environment by oocysts.
